# Interleukin-33 from Monocytes Recruited to the Lung Contributes to House Dust Mite-Induced Airway Inflammation in a Mouse Model

**DOI:** 10.1371/journal.pone.0157571

**Published:** 2016-06-16

**Authors:** Hiroki Tashiro, Koichiro Takahashi, Shinichiro Hayashi, Go Kato, Keigo Kurata, Shinya Kimura, Naoko Sueoka-Aragane

**Affiliations:** 1 Division of Hematology, Respiratory Medicine and Oncology, Department of Internal Medicine, Faculty of Medicine, Saga University, Saga, Japan; 2 Institute of Tokyo Environmental Allergy, Tokyo, Japan; French National Centre for Scientific Research, FRANCE

## Abstract

**Background:**

Interleukin-33 (IL-33) activates group 2 innate lymphoid cells (ILC2), resulting in T-helper-2 inflammation in bronchial asthma. Airway epithelial cells were reported as sources of IL-33 during apoptosis and necrosis. However, IL-33 is known to be from sources other than airway epithelial cells such as leukocytes, and the mechanisms of IL-33 production and release are not fully understood. The aim of this study was to clarify the role of IL-33 production by monocytes in airway inflammation.

**Methods:**

BALB/c mice were sensitized and challenged with a house dust mite (HDM) preparation. Airway inflammation was assessed by quantifying inflammatory cells in bronchoalveolar lavage (BAL) fluid, and IL-25, IL-33, and thymic stromal lymphopoietin (TSLP) levels in lung. Immunohistochemistry for IL-33 in lung sections was also performed. Ly6c, CD11b, and CD11c expression was examined by flow cytometry. Clodronate liposomes were used in the HDM-airway inflammation model to deplete circulating monocytes.

**Results:**

The IL-33, but not IL-25 or TSLP, level in lung homogenates was markedly increased in HDM mice compared to control mice. IL-33-positive cells in the lungs were identified using immunohistochemistry and were increased in areas surrounding bronchi and vasculature. Furthermore, IL-33 levels were increased in mononuclear cells derived from lungs of HDM mice compared to controls. The expression of Ly6c in mononuclear cells was significantly higher in HDM mice than in controls. Treatment with clodronate liposomes led to inhibition of not only inflammatory cells in BAL fluid, airway hyper reactivity and Th2 cytokines in lung, but also IL-33 in lung.

**Conclusion:**

IL-33 from monocytes recruited to the lung may contribute to the pathogenesis of HDM-induced airway inflammation.

## Background

Bronchial asthma is an airway inflammatory disease characterized by bronchoconstriction, airway hyper-responsiveness, and airway remodeling [1]. Airway eosinophilia, mediated mainly by T helper 2 (Th2)-type lymphocytes, has been reported to play an essential role in bronchial asthma [2,3].

Recently the genes encoding IL-33 and ST2 (also known as interleukin-1 receptor-like 1, IL-1RL1), have been identified as important factors for human asthma in several genome-wide association studies that included thousands of patients from diverse ethnic groups having different forms of asthma [4–6]. Other studies have suggested that early severe exacerbation of childhood asthma is closely correlated to the IL-33 gene [7]. Furthermore, high serum IL-33 was found to be related to severity of asthma [8], and IL-33 also caused airway remodeling in severe steroid-resistant asthma cases [9,10].

IL-33 is a member of the IL-1 family of cytokines and a specific ligand of the ST2/IL-1 receptor accessory protein (IL-1RAP) receptor complex [11]. IL-33 activates group 2 innate lymphoid cells (ILC2) and induces a large amount of Th2 cytokines such as IL-5 and IL-13 [12]. Th2 cytokines play a crucial role in bronchial asthma, which is characterized by eosinophilic airway inflammation and goblet cell hyperplasia [1,13,14]. The presence of IL-33 has been reported during necrosis or apoptosis of various cells including bronchial epithelial cells [15], alveolar type II cells [16], mast cells [17], dendritic cells [18], and vascular smooth muscle cells [19]. Previous studies have referred to bronchial epithelial cells as a major source of IL-33 in asthmatic airways. However, these findings remain controversial.

When pathogens or allergens invade their hosts, circulating monocytes mature into macrophages in specific organs [20]. Macrophages are classified into residential macrophages in tissues and recruited monocytes from the circulation [21,22]. Circulating monocytes, rather than residential alveolar macrophages, play a critical role in allergic airway inflammation [23].

Using a house dust mite (HDM)-induced airway inflammation mouse model, we demonstrated, in vivo and in vitro, the possibility that IL-33 from monocytes recruited to the lung played an important role.

## Materials and Methods

### Allergen and chemicals

Two batches of house dust mite (HDM) extract from *Dermatophagoides farina* (Der f) were provided by ITEA Inc. (Tokyo, Japan) as a lyophilized preparation of milled mites. Clophosome-A, liposomal clodronate and plain control liposomes were purchased from FormuMax Scientific Inc. (Palo Alto, CA, USA).

### Animals

Female BALB/c mice (Japan SLC Inc., Hamamatsu, Japan) aged 6–8 weeks were maintained at the Saga University animal facility under specific pathogen-free conditions. Animal experiments were undertaken following the guidelines for care and use of experimental animals of the Japanese Association for Laboratory Animals Science (1987) and were approved by the Saga University Animal Care and Use Committee.

### Protocol for HDM-induced airway inflammation and treatment of mice with clophosome-A and liposomal clodronate

Mice were sensitized intranasally with 25 μg HDM or vehicle on days 1, 8, and 15. Mice were challenged intranasally with 5 μg HDM on days 22, 23, and 24 [24]. Four hours after the final challenge, mice were euthanized by intraperitoneal injection with sodium pentobarbital. Serum, bronchoalveolar lavage fluid (BALF) and lung tissue were harvested for further analysis. Clodronate and control liposomes (150 μl / animal) were administered by intravenous injection on days 0, 7, 14, 21, 22, and 23. On the days when HDM was given, clodronate and control liposomes were given 30 minutes before the HDM inoculation.

### Collection of BALF

BALF samples were collected as described previously [24,25]. Briefly, a 20-gauge tube was inserted into the trachea and the lungs were lavaged with 1 ml of saline twice. The cell suspension was centrifuged at 100 × g for 5 min at 4°C. The total number of cells was counted using a hemocytometer. Cytospin samples were prepared from the cell suspension. Cell differentiation was determined by counting at least 300 leukocytes in samples stained with Diff-Quik (Siemens, Germany).

### H&E and PAS Histology

Histological examination was performed as previously reported. Lungs were fixed with 10% neutral buffered formalin (Wako, Japan) and embedded in paraffin. Lung sections were stained with Hematoxylin and Eosin (H&E) and periodic acid-Schiff (PAS) stains.

### Immunohistochemistry and Immunofluorescence

Paraffin sections of lung were deparaffinized in xylene and hydrated through graded alcohols. Sections were incubated overnight at 4°C with anti-IL-33 antibody or anti-normal goat IgG as the control (R&D Systems Inc., Minneapolis, MN, USA). Streptavidin-biotin amplification (Dako, Denmark) was carried out for 30 minutes, and hematoxylin was used to counterstain. For immunofluorescence, sections were incubated overnight at 4°C with anti-IL-33 antibody, and Northern Light 557 conjugated anti-goat IgG (R&D Systems Inc.) was used as the secondary antibody. FITC-conjugated anti-F4/80 was used to identify tissue macrophages (eBioscience, San Diego, CA). Fluorescent images were visualized using a bio-imaging navigator microscope system (Olympus, Tokyo, Japan).

### Preparation of lung homogenates

After bronchoalveolar lavage, the left lung was isolated and homogenized in 50 mM Tris buffered saline (pH 7.4) containing 1 mM EDTA, 1 mM PMSF, 1 μg/ml aprotinin, 1 μg/ml leupeptin, 1 mM Na_3_VO_4_, and 1 mM NaF. The lung homogenates were centrifuged at 10,000 × g for 15 min, and then supernatants were collected and stored at -80°C until required.

### Isolation of single cells from lung tissue

The peripheral lung tissue was cut into small pieces with scissors, transferred through 70 μm mesh and processed in digestion buffer which included deoxyribonuclease I (Invitrogen, Waltham, MA, USA) and collagenase type 2 (Worthington Inc., Lakewood, NJ). The remaining red cells were lysed using BD Pharm Lysis (BD Biosciences, San Jose CA), and single cells suspensions were obtained.

### Separation and analyzation of lung mononuclear cells from lung single cells

To separate lung mononuclear cells, the single cell suspensions of lung tissue were cultured at 37°C in a humidified atmosphere containing 5% CO_2_ for 24 hours. After 24 hours, culture medium was removed, plates were washed with PBS, and then adherent cells were harvested. More than 95% of the cells were recognized as monocytes by staining with Diff-Quik (Siemens, Germany). The cells were lysed by freezing and thawing to extract proteins and then frozen for further analysis.

### Quantification of cytokines using ELISA

IL-4, IL-5, IL-13, TNF-α, IL-1β, IL-6, IL-33, and IL-25 were measured using ELISA Kits (R&D Systems Inc.) according to the manufacturer’s instructions. All samples were tested in duplicate.

### Flow cytometry

Cells were stained with F4/80, CD11b, CD11c, CD45, CD115, and Ly6c (eBioscience, San Diego, CA, USA). Cells were collected on a FACS caliber flow cytometer (BD Bioscience, Franklin Lakes, NJ, USA) and analyzed with FlowJo 8.3.3 software.

### Airway hyper reactivity to methacholine

Mice were anesthetized by an intraperitoneal injection of pentobarbital and 18-gauge metal needle was inserted into exposed trachea. Airway hyper reactivity responses were measured by a forced oscillation technique (FlexiVent, SCIREQ, Montreal, Canada). Airway resistance (R) was investigated after exposures of increasing methacholine concentration.

### RNA extraction and real-time RT PCR

RNA was extracted from lungs, blood, and monocytes using the RNeasy® Protect Mini Kit (QIAGEN, The Netherlands). RNA quantity and quality were determined using a NanoDrop 1000A spectrophotometer (NanoDrop Products, Wilmington, DE, USA). RNA was reverse transcribed to cDNA, and Taqman gene expression assays were used to detect TSLP (Mm00498739-m1 Tslp), arginase-1 (Mm00475988-m1 arg1), Ym-1 (Mm0065789-mH Chil3), TNF (Mm00443260-g1 Tnf), IL-1β (Mm01336189-m1 Il1b), and 18S RNA (Mm03928990-g1 Rn18s). Messenger RNA expression levels were normalized using 18S RNA expression.

### Statistical analysis

The data are shown as mean ± standard deviation (SD). Analysis of variance (ANOVA) was used for multiple comparison of continuous variables. When there was a significant difference, the difference between each group was tested using Scheffe’s test. All tests were 2-sided, and significance was set at 0.05.

## Results

### IL-33 was significantly increased in lungs during HDM-induced airway inflammation

**Fig 1:**
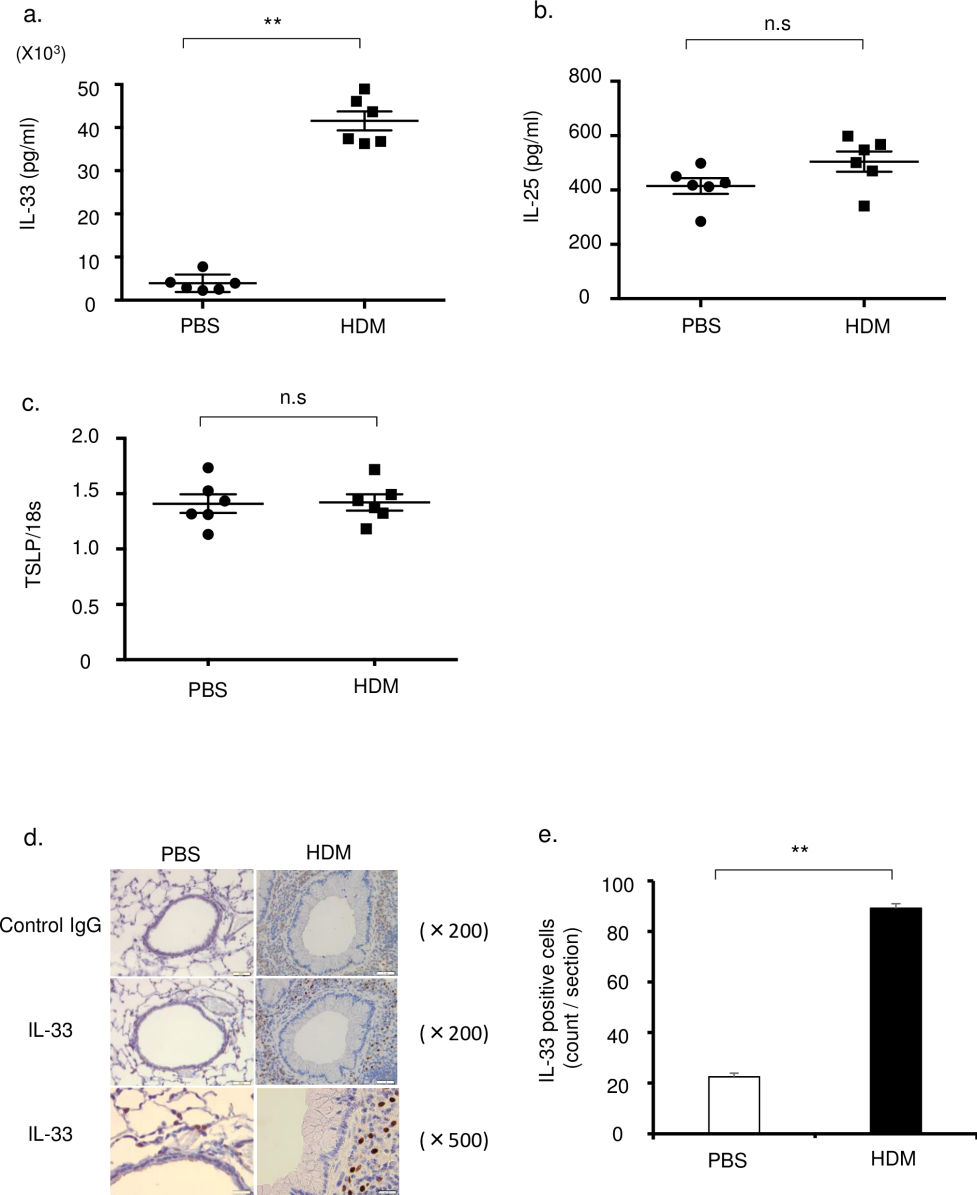
IL-33 was significantly increased in lungs of HDM mouse model. Concentrations of (a) IL-33, (b) IL-25 in the lung tissue were measured by ELISA (n = 6 in each group). (c) Thymic stromal lymphopoietin (TSLP) level in the lung tissue was measured by real-time RT-PCR assay (n = 6 in each group), because TSLP protein concentration was below the detection level. (d) Immunohistochemistry (IHC) examination of anti-normal goat IgG control and anti-IL-33 antibody in the lung section. Original magnifications were × 200 and × 500. (e) The number of IL-33 positive cells by IHC (6 sections were counted by two pulmonologists).

**Fig 2:**
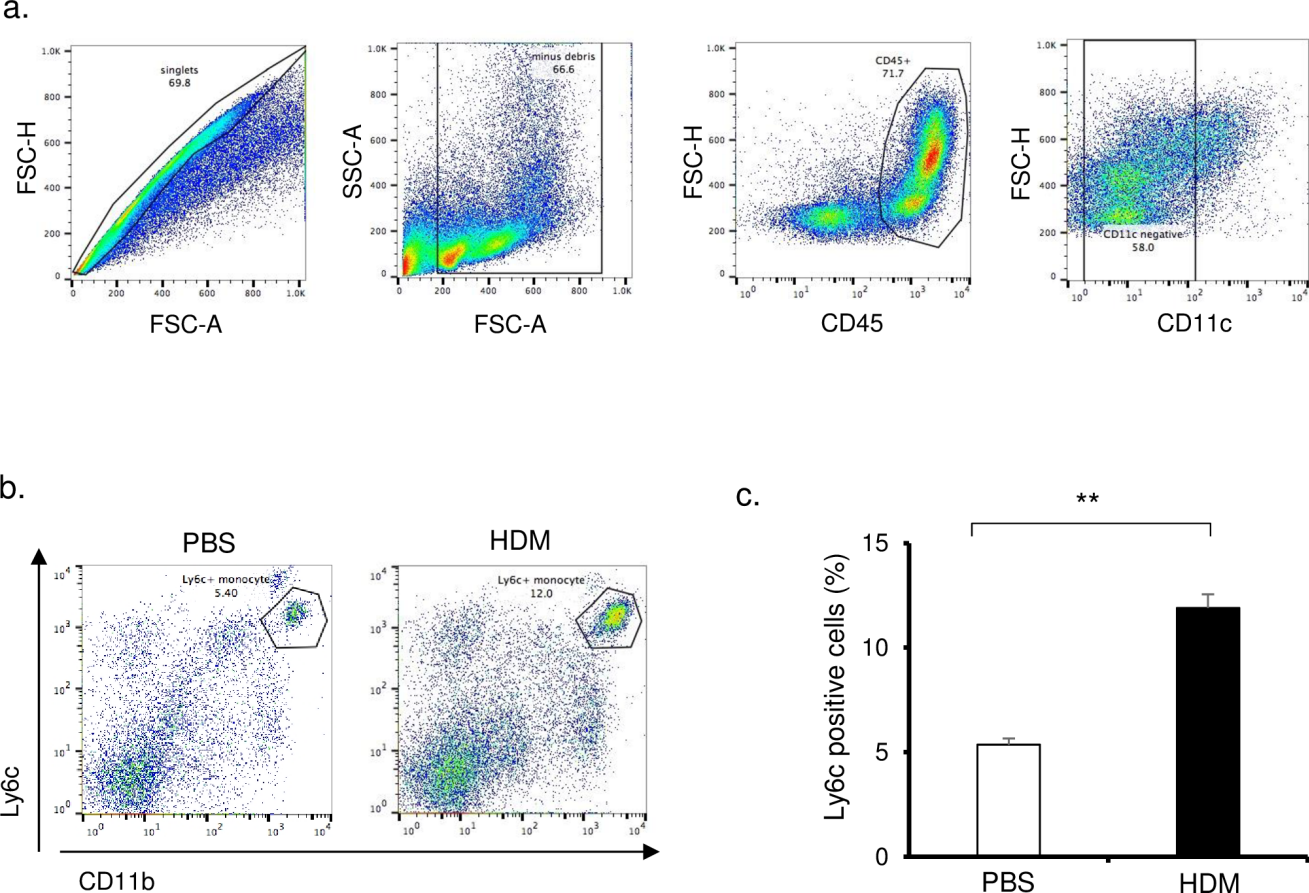
Flow cytometry analysis of Ly6c-positive monocytes in lungs of HDM mice and control mice. (a) Cells were identified from digested lungs, and after exclusion of doublets and debris, leukocytes were separated by CD45 staining. CD11c-negative cells were identified to investigate Ly6c-positive monocytes. (b) Flow cytometry analysis of Ly6c-positive monocytes (CD45^+^, CD11c^-^, CD11b^+^, Ly6c^+^) in lungs of HDM mice and control mice. (c) The percentage of Ly6c-positive monocytes in lung were compared in HDM mice and control mice (n = 6 in each group). **P<0.01

We investigated the involvement of cytokines in HDM-treated mice. The cytokines in BALF were undetectable by ELISA (data not shown). The concentration of IL-33 in lung tissue was significantly increased in HDM mice compared with control mice (Fig 1A). In contrast, IL-25 and TSLP in lung tissue were not increased in HDM mice (Fig 1B and 1C). Immunohistochemistry showed that IL-33-positive cells were located in the peribronchial regions of the lung. Bronchial epithelial cells, which were reported as a source of IL-33, were weakly stained (Fig 1D). The total positive cell counts in the IL-33-rich peribronchial regions were significantly increased in HDM mice compared with control mice (Fig 1E). The distribution and morphology of IL-33-positive cells by immunohistochemistry suggested that the cells were monocytes recruited from the bloodstream to the peribronchial regions. To identify monocytes in the lung, we examined the lung single cell suspensions by flow cytometry. After the exclusion of doublets and debris, leucocytes were separated using the pan-hematopoietic marker CD45. According to previous reports, Ly6c-positive monocytes were characterized by the expression of CD11b, Ly6c, and the absence of CD11c (Fig 2A) and alveolar macrophages by the expression of CD11c and the absence of CD11b (S1A Fig) [26]. Ly6c^+^ monocytes were increased in lungs of HDM mice compared with control mice (Fig 2B and 2C). In contrast, alveolar macrophages were not significantly increased (S1B Fig).

### Ly6c-positive lung mononuclear cells could be a source of IL-33 in vivo and ex vivo

**Fig 3:**
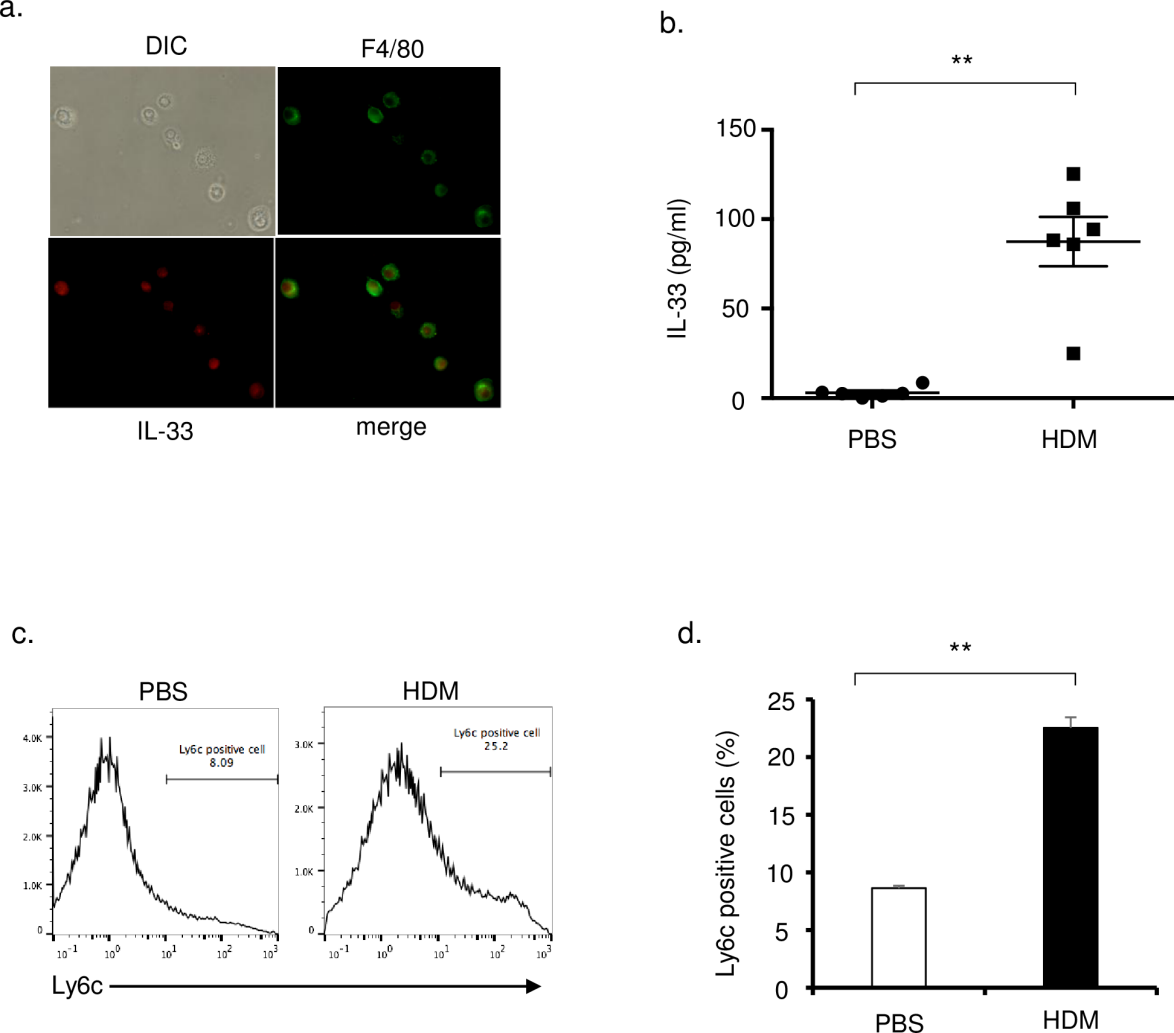
Lung mononuclear cells which positively expressed Ly6c are a source of IL-33. Lung mononuclear cells were collected from lung tissue as described in the text. Briefly, isolated single cells from lung tissue were resuspended at 2.0 × 10^6^ cells/ml in RPMI1640 medium supplemented with 10% FCS for 24 hours. Adherent cells were harvested and used as mononuclear cells in the experiments. (a) Immunofluorescence examination of lung mononuclear cells in HDM mice. Cells were stained with anti-F4/80 antibody (right upper panels) and anti-IL-33 antibody (left lower panels) and images were merged (right lower panels). Original magnification was × 800. (b) Lung mononuclear cells were lysed by freezing and thawing to extract protein. Concentrations of IL-33 in the lung mononuclear cells were measured by ELISA (n = 6 in each group). (c) Lung mononuclear cells were stained for Ly6c and analyzed by flow cytometery. Representative histograms of Ly6c^+^ cells in control mice and HDM mice are shown. (d) The percentage of Ly6c^+^ cells were counted by flow cytometery. **P<0.01.

To demonstrate that IL-33 was present within monocytes, we used mononuclear cells isolated from lung. These cells were confirmed to be more than 95% monocytes using Diff-Quik (data not shown). Using immunofluorescence, IL-33-positive cells were merged with F4/80-positive cells to identify monocytes (Fig 3A). IL-33 levels in these cells were increased in HDM mice compared with control mice (Fig 3B). To clarify whether these cells were recruited from blood, the number of Ly6c-positive cells was determined by flow cytometry and was found to be significantly increased in HDM mice (Fig 3C and 3D). These results suggest that monocytes recruited to the lung could be one of the sources of IL-33.

### Clodronate-liposomes attenuated HDM-induced airway inflammation and airway hyper reactivity

**Fig 4:**
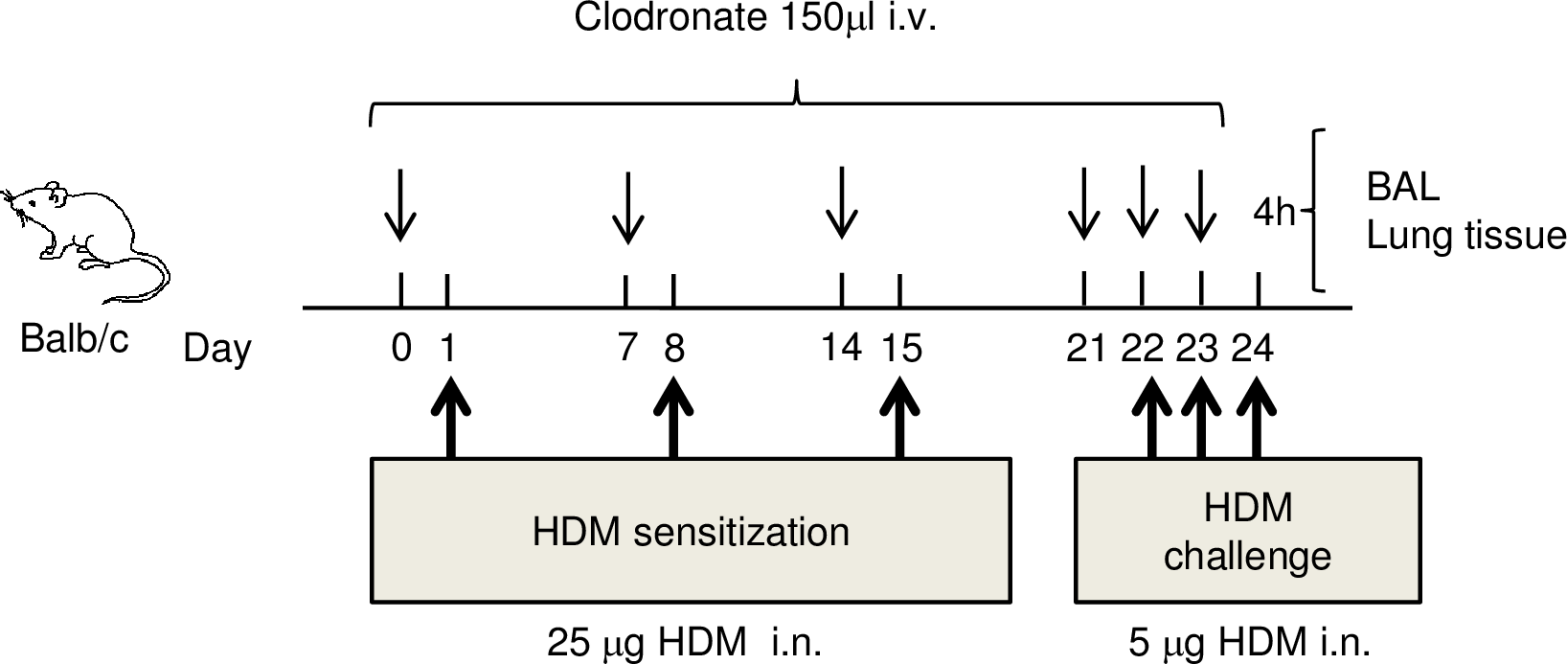
The protocols for HDM-induced airway inflammation and clodronate treatment of mice. Mice were sensitized intranasally with 25 μg HDM or vehicle on days 1, 8, and 15. Mice were challenged intranasally with 5 μg HDM on days 22, 23, and 24. Four hours later after final challenge, bronchoalveolar lavage (BAL) fluid and lung tissue were harvested for further analysis. Clophosome-A, liposomal clodronate, and plain control liposomes for clophosome (150μl / animal) were administered by intravenous injection on days 0, 7, 14, 21, 22, and 23. On the day when HDM was given, clodronate and control liposomes were administered 30 minutes before the HDM inoculation.

To clarify whether the IL-33-producing cells were monocytes recruited to the lung, we used clodronate-liposomes *in vivo*. Clodronate is a bisphosphonate compound which causes apoptosis by intracellular delivery to macrophages via liposomes. The effect is specific to phagocytic cells such as macrophages [27]. In mice with airway inflammation the administration route is critical for the type of macrophage depleted in the lung. If clodronate was administrated intratracheally, alveolar macrophages were depleted but not monocytes recruited from the bloodstream to the tissue. In contrast, if clodronate was administrated intravenously, monocytes from the blood were depleted but not alveolar macrophages [23]. We investigated the effect of clodronate administrated intratracheally to HDM-induced airway inflammation mice (Fig 4). More than 90% of monocytes were depleted after 24 hours and numbers were unchanged after 48 hours in the spleen. In bone marrow, 70% of monocytes were depleted after 24 hours of administration, but numbers were recovered after 48 hours (S2 Fig). Lymphocytes in the spleen were unchanged after administration of clodronate (data not shown).

**Fig 5:**
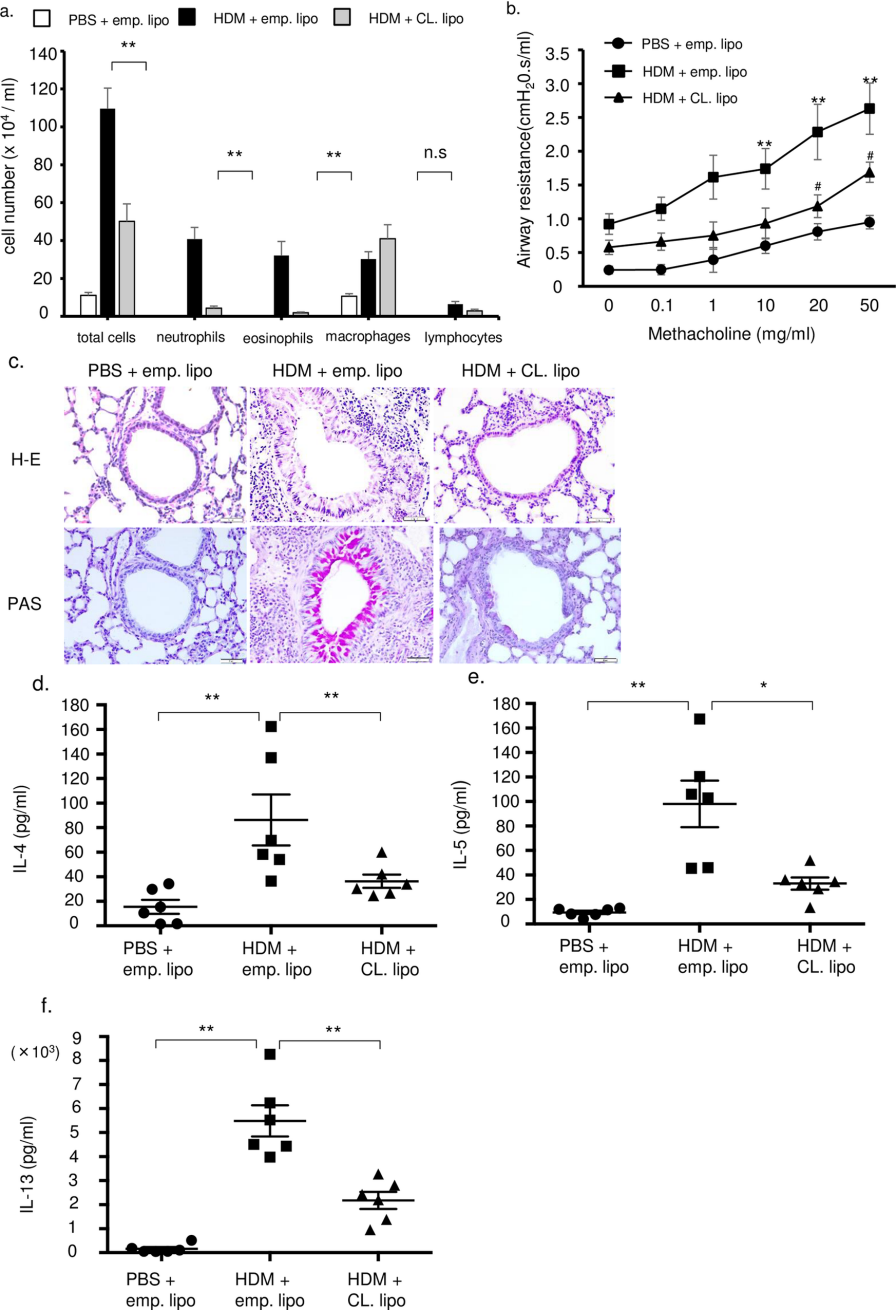
Clodronate-liposome attenuated the HDM induced airway inflammation, airway hyper reactivity and production of Th2 cytokines. (a) Total and differential cell counts in bronchoalveolar lavage fluids (n = 6 in each group). (b) Airway hyper reactivity was measured by assaying airway resistance during graded concentration of methacholine (n = 6 in each group). ^#^p<0.05 HDM+emp. lipo group compared with PBS+emp. lipo group. ^##^p<0.05 HDM+CL. lipo group compared with HDM+emp. lipo group. (c) Histological examination of airway inflammation. Sections were stained with H&E (upper panels) or PAS (lower panels). Original magnification was × 200. Empty liposome control (emp. lipo) and clodronate liposome (CL) results are shown. Concentrations of (d) IL-4, (e) IL-5, and (f) IL-13 in the lung tissue were measured by ELISA (n = 6 in each group). **P<0.01, *P<0.05

**Fig 6:**
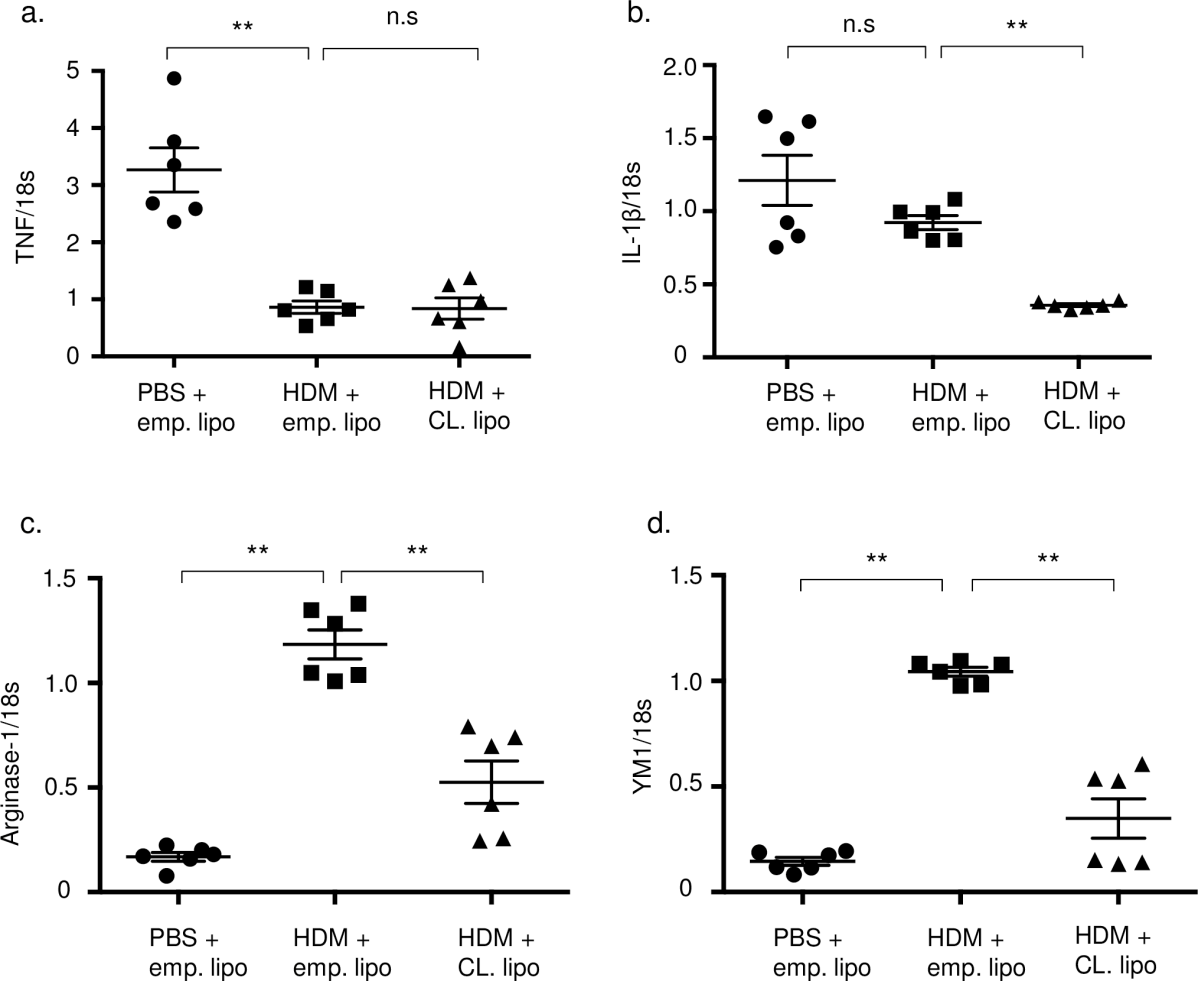
Lung mononuclear cells expressed M2 markers but not M1 markers. The expression of TNF (a), IL-1β (b), arginase-1 (c), and YM-1 (d) levels in the lung mononuclear cells was measured by real-time RT-PCR assay.

Total and inflammatory cells including neutrophils and eosinophils, but not macrophages, in BALF were significantly decreased after clodronate treatment (Fig 5A). Airway hyper reactivity of HDM mice was significantly increased compared with control mice and this elevation was significantly attenuated by administration of clodronate (Fig 5B). Inflammatory cells and goblet cell hyperplasia were also inhibited by clodronate treatment compared with untreated HDM mice (Fig 5C). Th2 cytokines such as IL-4, IL-5, and IL-13 in lung tissues were also inhibited in clodronate-treated mice (Fig 5D–5F). However, TNF-α, IL-1β and IL-6 were not attenuated by administration of clodronate (S3A–S3C Fig).We next examined TNF and IL-1β expression as markers of M1 macrophages and arginase-1 and Ym1 as markers of M2 macrophages. Interestingly, M2 markers were decreased by clodronate treatment compared with HDM mice, while M1 markers were not affected by clodronate treatment (Fig 6A–6D). These results suggest that HDM-induced airway inflammation depends on recruited monocytes, which polarize the response toward the M2 phenotype.

### Monocytes recruited to lung are a source of IL-33

**Fig 7:**
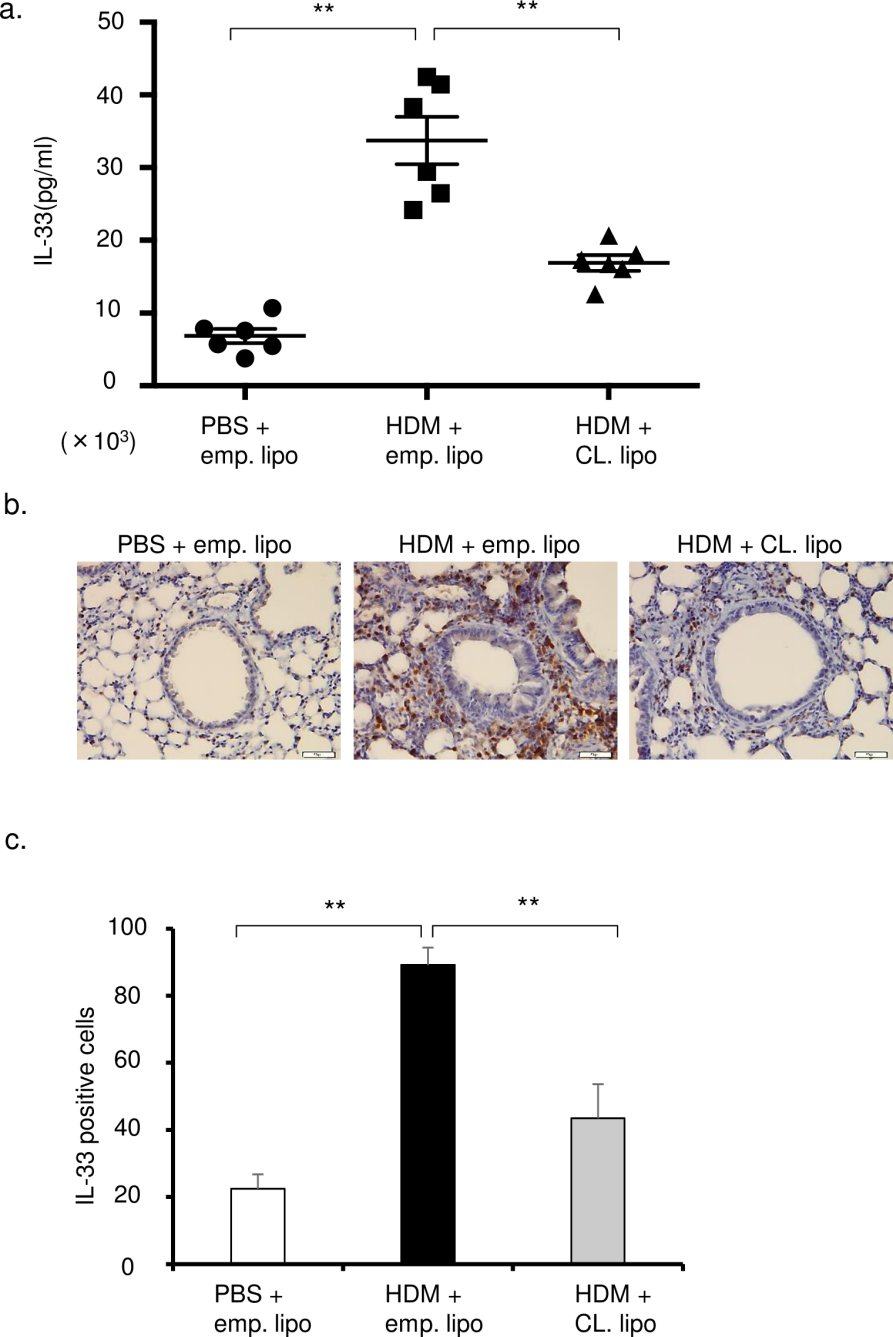
Clodronate attenuated the IL-33 in lungs of HDM mice. (a) Concentrations of IL-33 in the lung tissue were measured by ELISA in 3 groups (intranasal PBS and intravenous empty liposomes, HDM and empty liposomes, HDM and clodronate liposomes: n = 6 in each group). (b) Immunohistochemistry (IHC) examination of IL-33 in the lung sections. Original magnification was × 200. (c) IL-33-positive cell counts by IHC (6 sections were counted by two pulmonologists). **P<0.01.

IL-33 in lung tissue was significantly decreased in clodronate-treated mice compared with HDM mice (Fig 7A). IL-33-positive cells in immunohistochemistry were clearly decreased in clodronate-treated mice (Fig 7B). The number of IL-33-positive cells was significantly decreased in the peribronchial regions of clodronate-treated mice compared with HDM mice (Fig 7C). These results suggest that clodronate attenuated IL-33 in HDM mice and that recruited monocytes, but not residential alveolar macrophages, were the sources of IL-33.

## Discussion

The results of this study demonstrated that monocytes recruited to lung are a potent source of IL-33 and enhance Th2 airway inflammation induced by HDM. Immunohistology showed the presence of intranuclear IL-33 in pulmonary monocytes. IL-33 was released from pulmonary mononuclear cells, which were shown to be mostly monocytes. Depletion of circulating monocytes by clodronate liposomes led to inhibition of HDM-induced airway inflammation, meaning that recruited monocytes were essential for airway inflammation. To our knowledge, this is the first report to identify that IL-33 from inflammatory monocytes recruited to the lung contributes to airway inflammation.

IL-33 has emerged as a danger signal that is rapidly released from various cells by cellular damage or stress [28]. Biologically active full length IL-33 was released into the extracellular space after cell necrosis or mechanical injury [29]. IL-33 induces the production of Th2 cytokines such as IL-5 and IL-13 through activation of ILC2s, leading to allergic inflammation [11]. Bronchial epithelial cells were reported as a major source of IL-33 in previous studies [30,31]. In airway diseases, IL-33 was shown to be released into the airways after stimulation with influenza viruses [32], alternaria [15], and papain [33]. These responses occurred in the early phase after stimulation because IL-33 concentration reached a maximum at 6 to 24 hours. Thus IL-33 is released via the innate immune system as an initial mediator in allergic airway inflammation following attack from exogenous allergens and pathogens [14]. In this study, IL-33 in BALF was not detected in our mouse model, while markedly increased IL-33 was noted in lung homogenates from mice challenged with HDM. We found that IL-33 stained cells that shared morphological and immunological characteristics with macrophages were present around the bronchovascular areas (Fig 1D). We were able to detect only a few bronchial epithelial cells stained with IL-33 in the immunohistological findings. We considered the possibility that IL-33 within bronchial epithelial cells might have been released in an earlier phase of HDM sensitization.

Macrophages are the most abundant pulmonary immune cells and are responsible for antigen and allergen recognition and presentation [34,35]. Alveolar macrophages in patients with severe asthma were decreased in LPS-responsiveness that is manifested by defective apoptotic cell uptake and reduced secretion of inflammatory mediators [36]. Pulmonary macrophages have also been reported to be associated with severe asthma characterized by glucocorticoid-resistance and airway remodeling [10]. Li and colleagues reported that macrophages are involved in glucocorticoid-resistant airway hyperresponsiveness (AHR) through a MyD88-dependent mechanism [37].

We demonstrated that clodronate treatment inhibited airway inflammation and IL-33 release, which indicated that inflammatory monocytes recruited to the lung had a crucial role in HDM-induced airway inflammation. Ly6c has been reported as a marker of inflammatory monocytes in blood that cause tissue inflammation [20,38,39]. We showed that Ly6c-positive monocytes, but not alveolar macrophages, were increased in lungs of HDM mice. These data suggest that recruited monocytes were more important in HDM-induced airway inflammation than alveolar macrophages.

We also showed that the expression of Ly6c in lung mononuclear cells was increased in HDM mice compared with control mice (Fig 2C and 2D). These results suggest that monocytes recruited to lung were one of the sources of IL-33.

Macrophages have been classified into M1 and M2 subtypes based on their expression of cytokines, especially chemokines, and other specific markers [40]. M1 macrophages are characterized by a pro-inflammatory phenotype that promotes the Th1 immune response and anti-tumor activity. M2 macrophages play a role in regulatory functions in tissue repair, remodeling, and promotion of the Th2 immune response [41–43]. We showed that M2 macrophage markers decreased following clodronate treatment compared to HDM mice, suggesting that recruited monocytes were involved in HDM-airway inflammation. These data are consistent with previously reported functions of M2 macrophages in allergic disease [40].

We used a first generation bisphosphonate, clodronate, in liposomes, to deplete macrophages in this study [27]. A large dose of a second generation bisphosphonate, alendronate, attenuated eosinophilic airway inflammation induced by ovalbumin in mice [44]. In this study, we performed airway sensitization and challenge with HDM in mice. Our data indicated that circulating cells such as monocytes had a crucial role in airway inflammation, suggesting that bronchial asthma involves not only the airways but also the systemic circulation. Targeted therapy against systemic monocytes might be a potential treatment for severe, treatment-resistant asthma. It is expected that new agents with higher specificity for monocytes will be developed.

In conclusion, IL-33 derived from monocytes recruited to the lung may contribute to HDM-induced airway inflammation in vivo and in vitro. This study describes, to our knowledge for the first time, mechanisms of IL-33-related airway inflammation through recruited monocytes.

## Supporting Information

S1 Fig Flow cytometry analysis of alveolar macrophages were not increased in lungs of HDM mouse model. Lung cells from control and HDM mice were separated by flow cytometry and examined. (a) Lung single cells were examined after the exclusion of doublets and debris and CD45^+^ cells were isolated. (b) Alveolar macrophages (CD45^+^, CD11b^-^, CD11c^+^) in lungs of HDM and control mice were compared.
(TIF)

S2 Fig Depletion of bone marrow monocytes and splenic monocytes after administration of clodronate lipopsome in a mouse model. (a) Depletion of CD11b^+^ F4/80^+^ bone marrow monocytes and CD115^+^ F4/80^+^ spleen monocytes at 24 hours after intravenous administration of clodronate liposomes compared to mice treated with control liposomes, analyzed by flow cytometry. Bone marrow cells were isolated from femur. Spleen was digested and separated into single cells. (b) The kinetics and compartment-specific depletion of monocytes after clodronate liposome administration, determined by flow cytometry. Solid line depicts percentage of CD11b^+^ F4/80^+^ bone marrow monocytes. Dashed line depicts percentage of CD115^+^ F4/80^+^ splenic monocytes. Data points are means of 3 mice at each time point.
(TIF)

S3 Fig Clodronate did not attenuate the TNF-α, IL-1β and IL-6 in lung of HDM mice. Concentrations of (a) TNF-α, (b) IL-1β and (c) IL-6 in lung tissue were measured by ELISA (n = 6 in each group).
(TIF)
