# Supplementary figures and images for: Interleukin-33 from Monocytes Recruited to the Lung Contributes to House Dust Mite-Induced Airway Inflammation in a Mouse Model
Source: PLoS One. 2016 Jun 16;11(6):e0157571. doi: 10.1371/journal.pone.0157571 (PMC4910993; doi:10.1371/journal.pone.0157571)

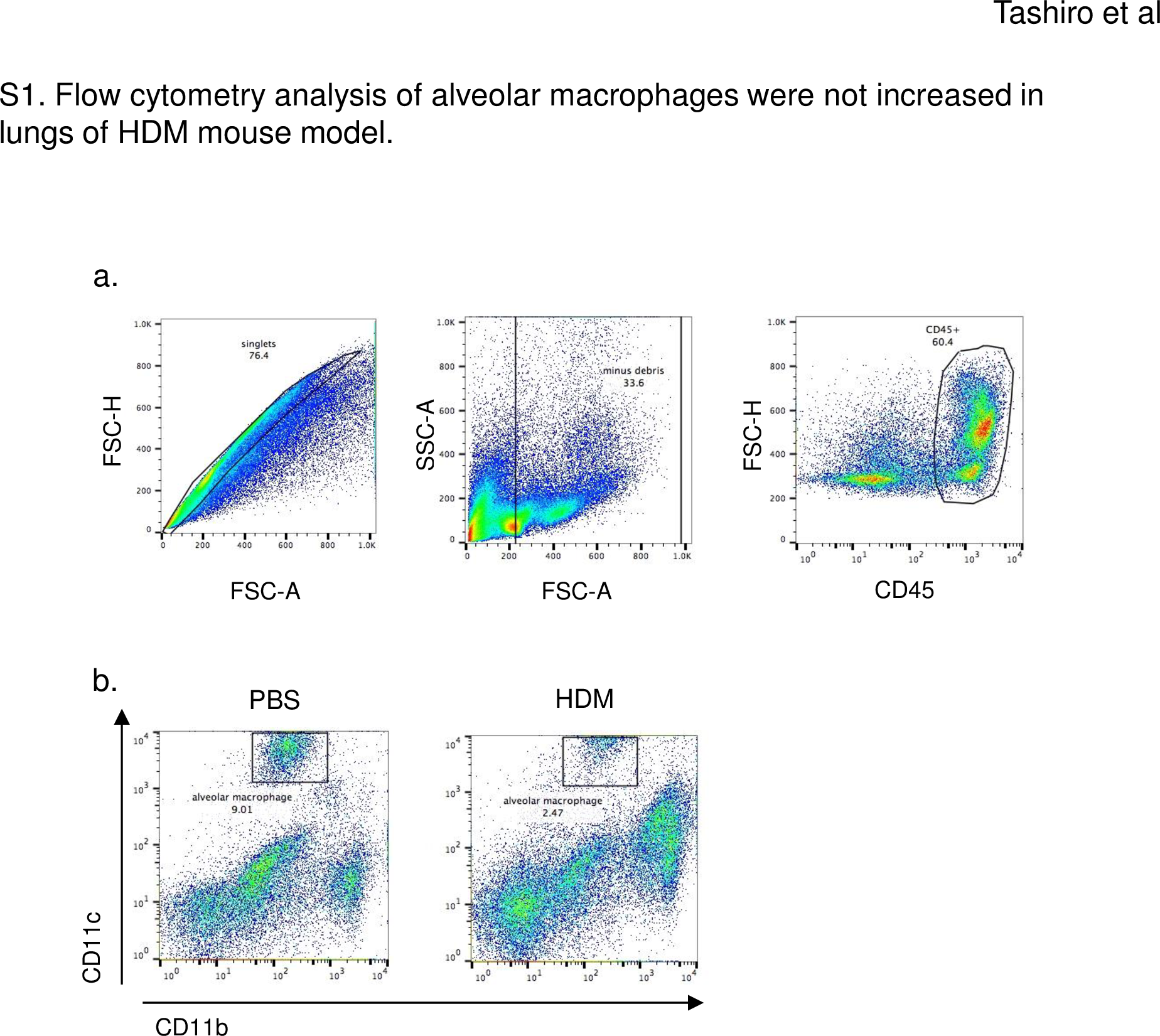

Supplement: S1 Fig — Lung cells from control and HDM mice were separated by flow cytometry and examined. (a) Lung single cells were examined after the exclusion of doublets and debris and CD45+ cells were isolated. (b) Alveolar macrophages (CD45+, CD11b-, CD11c+) in lungs of HDM and control mice were compared. (TIF) [file pone.0157571.s001.tif]

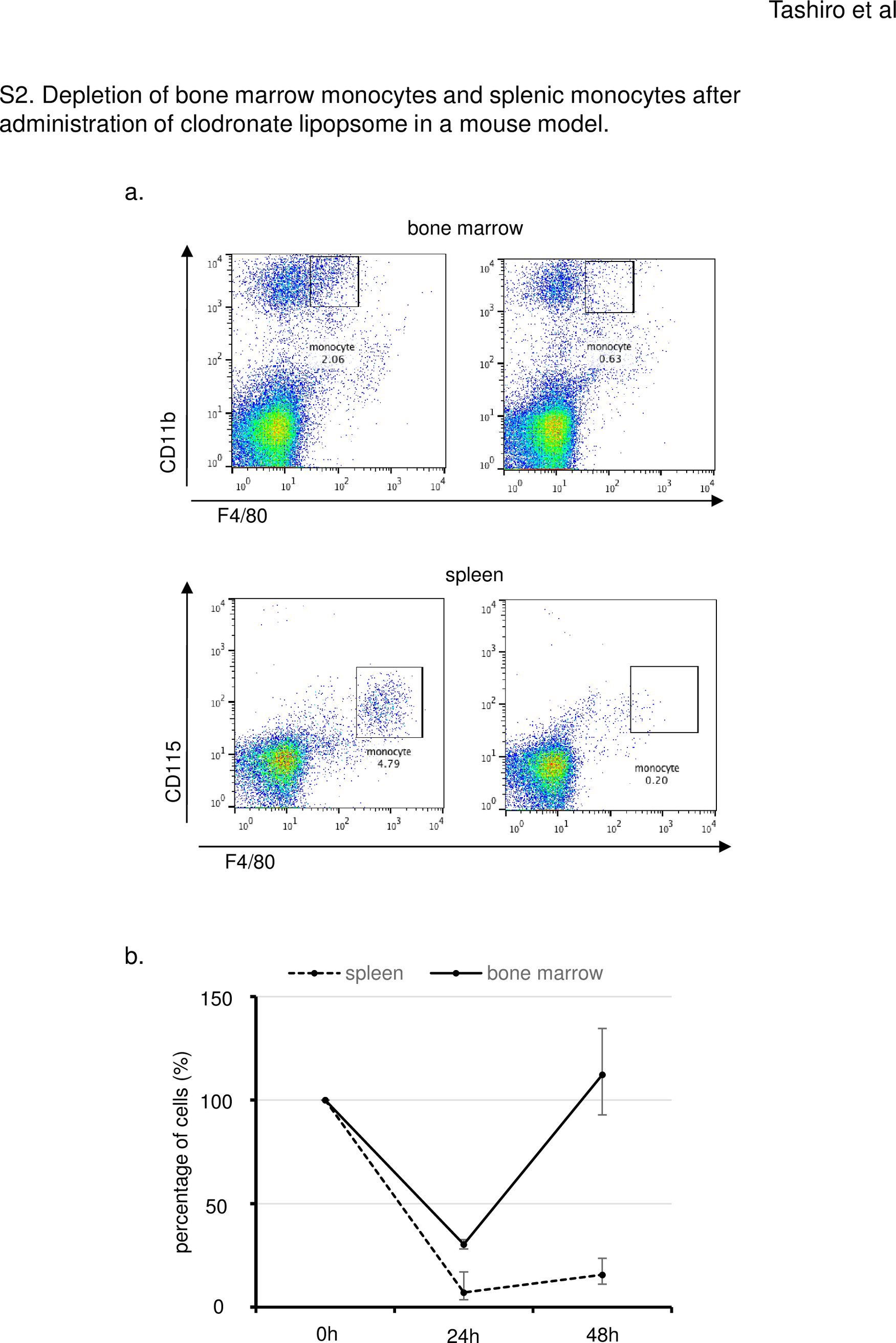

Supplement: S2 Fig — (a) Depletion of CD11b+ F4/80+ bone marrow monocytes and CD115+ F4/80+ spleen monocytes at 24 hours after intravenous administration of clodronate liposomes compared to mice treated with control liposomes, analyzed by flow cytometry. Bone marrow cells were isolated from femur. Spleen was digested and separated into single cells. (b) The kinetics and compartment-specific depletion of monocytes after clodronate liposome administration, determined by flow cytometry. Solid line depicts percentage of CD11b+ F4/80+ bone marrow monocytes. Dashed line depicts percentage of CD115+ F4/80+ splenic monocytes. Data points are means of 3 mice at each time point. (TIF) [file pone.0157571.s002.tif]

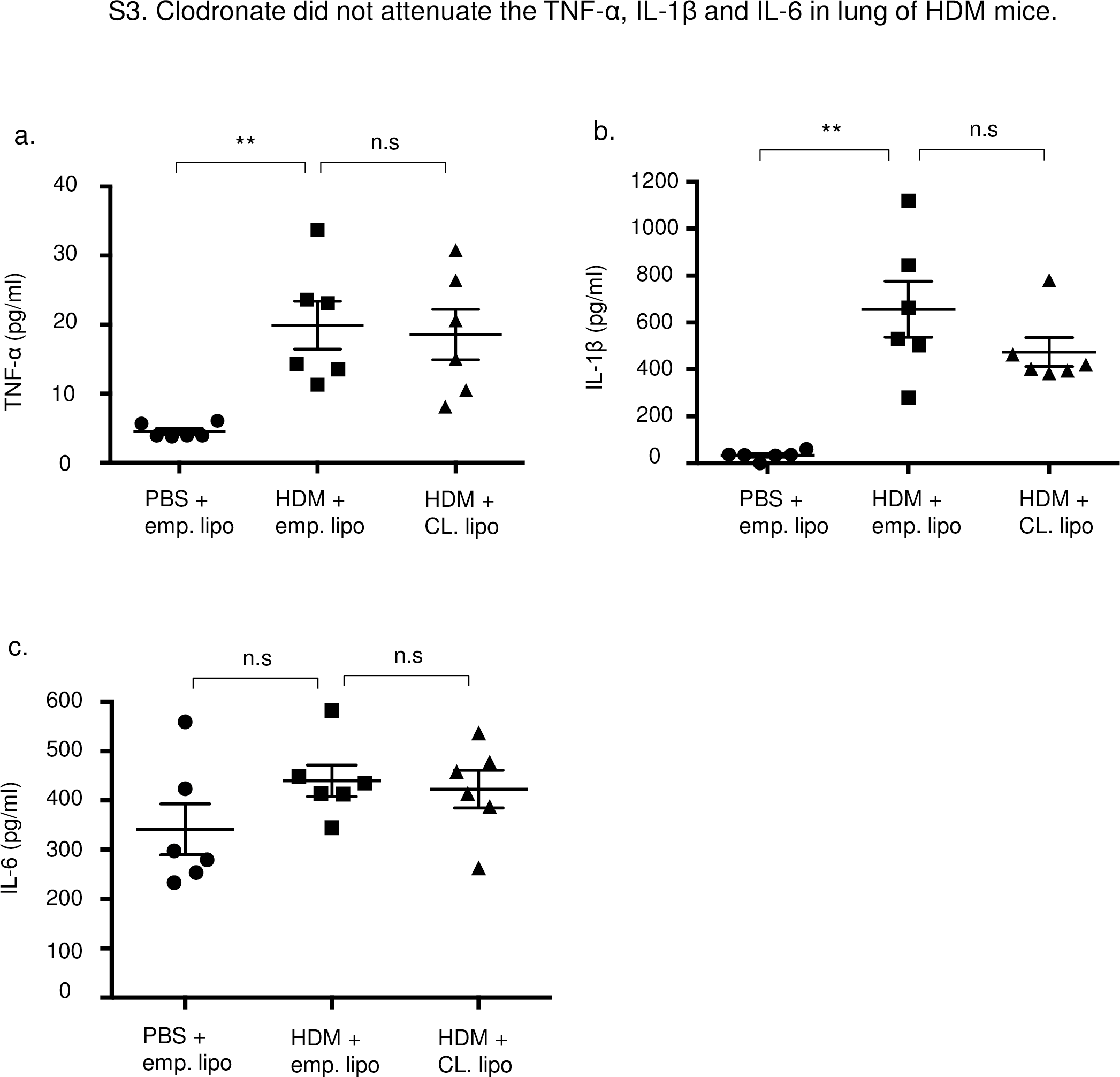

Supplement: S3 Fig — Concentrations of (a) TNF-α, (b) IL-1β and (c) IL-6 in lung tissue were measured by ELISA (n = 6 in each group). (TIF) [file pone.0157571.s003.tif]
